# 

**Published:** 1886-07

**Authors:** 


### SOUTHERN DENTAL ASSOCIATION.

The eighteenth annual meeting of the Southern Dental Association will convene in Nashville, Tenn., Tuesday the 27th day of July, 1886, at 10 o'clock A. M., and continue four days.

The Association will hold its sessions in the Lecture Hall of Watkins Institute, south-west corner of Church and High streets. This Hall is equi-distant from the two leading hotels, is large, well ventilated and easy of access (no stairs).

A most cordial invitation is extended to the profession generally to be present, and to affilliate with this the representative body of dentists of the South.

The Committee has secured the following hotel rates:

| | |
|---|---|
| Maxwell House, per day | $2 00 |
| Nicholson House, per day, (single) | 2 00 |
| Nicholson House, per day, (double) each | 1 50 |

It is very desirable, and those expecting to attend are respectfully urged to engage their rooms in advance by writing at once, stating time of arrival. On registering indicate to clerk, by personal card or otherwise, that you are attending the Southern Dental Association.

The railroads, under the control of the Southern Passenger Committee, through Commissioner M. Slaughter, have agreed to carry dentists, attending this meeting, over their roads for one and one-third fare for round trip, which can be had from local ticket agents, upon presentation of a *certificate, previously obtained* from Dr. R. A. Holliday, of Atlanta, Ga.

The trunk lines will also give reduced rates, and *certificates* can be had of Mr. J. W. Selby, care S. S. White, Dental Manufacturing Company, Philadelphia, Pa.

Greatly reduced round-trip tickets, from Nashville to the Mammoth Cave, can be had, and arrangements will be made for an excursion at the close of the session.

Those having prepared papers or essays to read, should at once notify the Committee of Arrangements that it may be assigned a place on the programme.

Quite a number of prominent men have signified their intention to be present, and have consented to clinic. One day will be set apart for clinics, and this promises to be quite a valuable feature.

Special arrangements have been made for the demonstration of the new Crown and Bridge-Work, Dr. W. Storer How will be in constant attendance to give any explanation desired of the methods and instruments.

The display of Dentists' supplies will embrace everything of value required in dental practice, including the latest and most improved appliances.

The Committee will endeavor to furnish pleasant and agreeable entertainment for the evenings during the session of the Association.

The programme for the week, which will contain many interesting features, will be distributed at the opening.

The officers, members, and all others, are respectfully requested to be present promptly on the morning of the first day. Every indication is that there will be a large attendance, and a most profitable meeting.

W. H. MORGAN,
J. C. MORRISON,
R. R. FREEMAN,
*Committee of Arrangements.*

ADDRESS BEFORE THE ALUMNI ASSOCIATION OF THE DEPARTMENT OF MEDICINE AND SURGERY OF THE UNIVERSITY OF MICHIGAN.—By Charles J. Lundy, M. D.

### *To Readers and Correspondents!*

Communications intended for publication, and exchange journals and papers should be addressed to the EDITOR OF THE AMERICAN JOURNAL OF DENTAL SCIENCE, 259 N. Eutaw St., Baltimore, Md. All letters relating to the business of the JOURNAL, or containing cash remittances, to be directed to Messrs. SNOWDEN & COWMAN. Articles for publication should be received by the editor at least three weeks previous to the first month on which the number in which it is designed for them to appear, is issued.

☞ The AMERICAN JOURNAL OF DENTAL SCIENCE will contain fifty pages of reading matter in each number, making a volume of about *Six Hundred* pages,

EXCLUSIVE OF ADVERTISEMENTS.

